# A novel investigation of *NANOG* and *POU5F1* associations in the pluripotent characterization of ES-like and epiblast cells

**DOI:** 10.1038/s41598-024-75529-4

**Published:** 2024-10-15

**Authors:** Mehdi Mehdinezhad Roshan, Hossein Azizi, Kiana Sojoudi

**Affiliations:** 1 Faculty of Biotechnology, Amol University of Special Modern Technologies, P.O.Box: 46168-49767, Amol, Iran; 2 Department of Reproductive Biology, Faculty of Advanced Medical Sciences, Tabriz University of Medical Sciences, Tabriz, Iran

**Keywords:** NANOG, POU5F1, ES-like cells, Epiblast cells, Spermatogenesis, PPI network, Cell biology, Computational biology and bioinformatics, Developmental biology, Stem cells

## Abstract

The transcription factors NANOG and POU5F1 (OCT4) play crucial roles in maintaining pluripotency in embryonic stem (ES) cells. While their functions have been well-studied, the specific interactions between NANOG and POU5F1 and their combined effects on pluripotency in ES-like and Epiblast cells remain less understood. Understanding these associations is vital for refining pluripotent stem cell characterization and advancing regenerative medicine. In this matter, we investigated the associations between NANOG and POU5F1 in maintaining pluripotency in ES-like and Epiblast cells and how these interactions contribute to the distinct pluripotent states of these cells. In the present paper, we examined the pattern of NANOG expression by the immunocytochemical method in embryonic stem-like (ES-like) cells and compared it with its expression pattern in embryonic stem cells (ESCs). Similarly, we examined the expression pattern of POU5F1 in ES-like cells, ESCs, and epiblast cells and compared the expression pattern of these two genes with each other. On the other hand, using Fluidigm Biomark system analysis, we compared the amount of NANOG mRNA in these three cell lines and differentiated and undifferentiated Spermatogonial stem cells in several passages. Microscopic observations indicated the cytoplasmic expression of NANOG in the considered cells; moreover, they showed a similar expression pattern of NANOG with POU5F1 in the experimented cells. It has also been suggested that the more limited the cell’s pluripotency, the lower the expression of these two genes. However, the decrease in NANOG expression is less than that of POU5F1. Fluidigm real-time RT-PCR analysis also confirmed these results. During the experimental process, protein-protein (PPI) network analysis shows a significant association of NANOG with other stem cell proteins, such as POU5F1. Our findings reveal distinct yet overlapping roles of NANOG and POU5F1 in maintaining pluripotency in ES-like and Epiblast cells. The differential binding patterns and functional interactions between these factors underscore the complexity of pluripotency regulation in different stem cell states. This study provides new insights into the molecular mechanisms governing pluripotency and highlights potential targets for enhancing stem cell-based therapies.

## Introduction

Pluripotency, the ability of a cell to differentiate into any cell type within an organism, is a fundamental property of embryonic stem (ES) cells^1,2^. This characteristic is governed by a complex network of transcription factors, among which NANOG and POU5F1 (OCT4) play pivotal roles^3^. NANOG is known for its critical function in maintaining the undifferentiated state of ES cells, while POU5F1 is essential for the self-renewal and pluripotency of these cells^4^. Despite extensive studies on their individual contributions, the precise nature of their interactions and the combined effects on pluripotency across different stem cell types remain incompletely understood^5,6^.

Recent advances in stem cell biology have revealed that pluripotency is not a uniform state but a spectrum, with subtle differences in molecular signatures depending on the cell type and developmental stage^7,8^. For instance, ES cells derived from the blastocyst’s inner cell mass (ICM) represent a “naive” pluripotent state characterized by a specific transcriptional profile and high levels of NANOG and POU5F1^7,9^. In contrast, epiblast cells, which emerge slightly later in development, exhibit a “primed” pluripotent state with distinct molecular characteristics, reflecting their readiness for lineage commitment^10,11^.

Given these disparities, it is essential to understand how NANOG and POU5F1 interact within and between these pluripotent states^12^. This understanding could provide insight into the underlying mechanisms that govern the transition from a naive to a primed state and the maintenance of pluripotency itself^13^. Furthermore, elucidating these interactions could have significant implications for regenerative medicine, where precise modulation of the pluripotent state of stem cells is crucial for their application in cell-based therapies^14^.

Over the past few years, various research groups have successfully derived embryonic-like stem cells from spermatogonial stem cells through the modulation of Nanog, Pou5F1, Sox2, Dazl, and c-Myc gene expression^15–17^. Notably, Nanog has played a crucial and prominent role^7^, and gaining a deeper understanding of its mechanisms and interactions could offer valuable insights into regenerative medicine and stem cell biology.

In this study, we investigate the associations between NANOG and POU5F1 in the pluripotent characterization of ES-like and Epiblast cells. We aim to explore how these transcription factors collaborate to regulate pluripotency and how their interactions differ between these two cell types. Through a combination of molecular and functional assays, we seek to unravel the complexities of pluripotency regulation and provide new insights into the molecular underpinnings of stem cell biology.

## Result

### Immunocytochemistry of NANOG expression in ESCs, ES-like cells, and epiblast cells

**Fig. 1:**
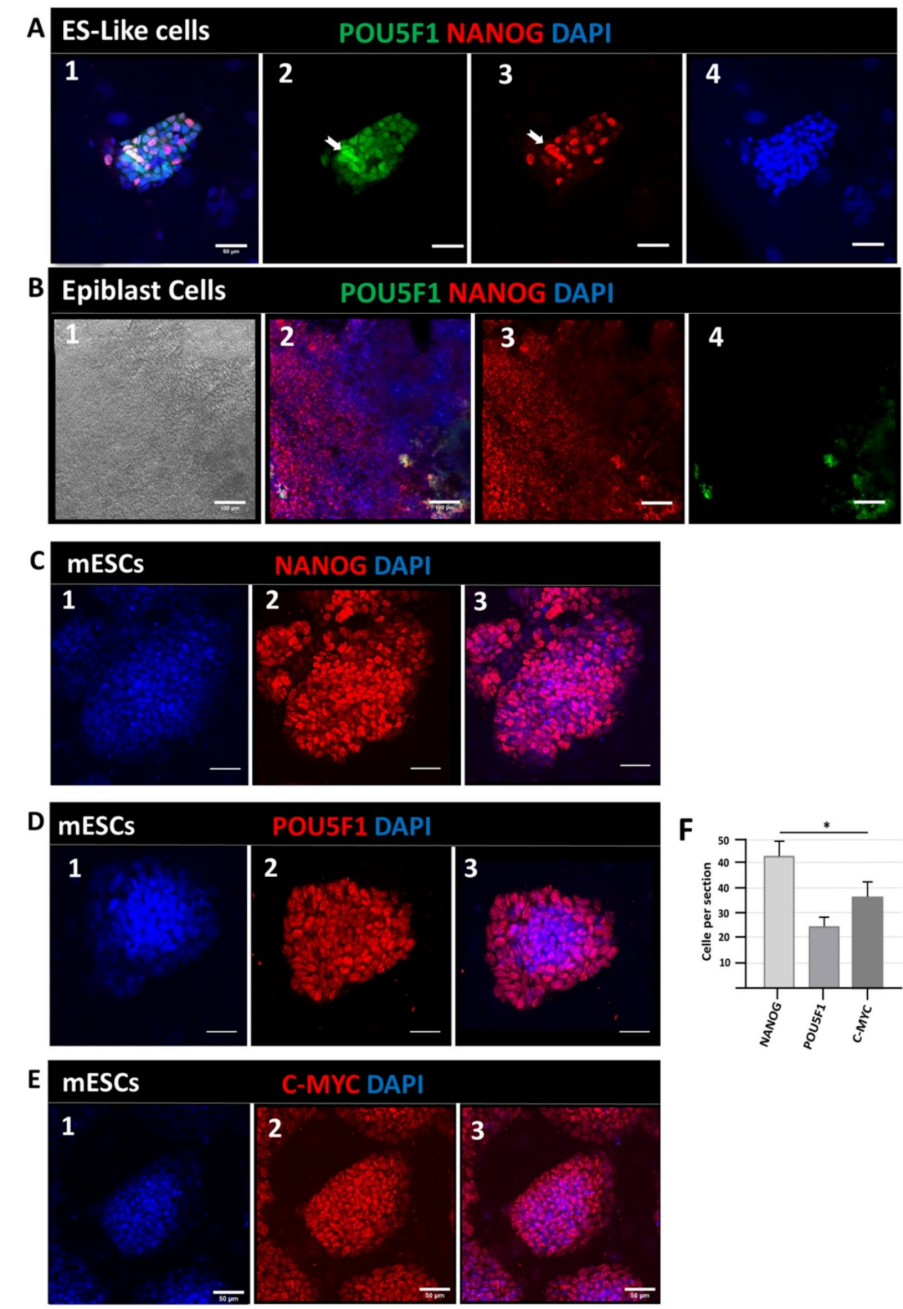
Immunocytochemical analysis of NANOG, POU5F1, and C-MYC expression patterns in ES-like cells (**A**), Epiblast cells (**B**), and embryonic stem cells (**C**–**E**). **A** (1) Representation of the merged images (2) Green fluorescence shows POU5F1 which has a high expression level in ES-like cells; (3) Red fluorescence shows the NANOG expression pattern, which has a high and the same expression pattern as POU5F1, and also has both cytoplasmic and nuclear expression; (4) Blue for 4’, 6-diamidino-2-phenylindole (DAPI) was used to stain cell nuclear. **B** (1) Bright-field image represents the morphology of the epiblast cells population; (2) Representation of the merged images with DAPI; (3) Red fluorescence shows NANOG expression; (4) Green fluorescence shows POU5F1 expression in Epiblast cells, which is lower than NANOG expression, but both show reduced expression in epiblast cells compared to ES-like cells and ESCs. **C** (1) Blue DAPI (non-specific core staining); (2) Red fluorescence shows the high expression of NANOG in the ESCs, which displayed nuclear and cytoplasmic staining; (3) Representation of the merged image with DAPI. **D** (1) Blue DAPI (non-specific core staining); (2) POU5F1 expression in ESCs (3) merged image with DAPI. **E** (1) Blue DAPI (non-specific core staining); (2) C-MYC expression in ESCs; and (3) merge image with blue DAPI (scale bar: 50 μm). The number of NANOG^+^, POU5F1^+^, and C-MYC^+^ cells per section *p* ≤ 0.05.

As the first step in this experimental study, immunocytochemical analysis was used to compare the expression pattern of NANOG and POU5F1 (OCT4), one of the essential markers of pluripotency, in ES-like cells, ESCs, and Epiblast cells. As apparent in Fig. 1, NANOG and POU5F1 showed the same expression pattern in the ES-like cell population (Fig. 1A). NANOG cytoplasmic expression is also observed in ES-like cells (Fig. 1A3).

We have continued the ICC analysis of NANOG and compared its expression with POU5F1 in Epiblast cells (Fig. 1B). Microscopic images (Fig. 1) show a decrease in POU5F1 expression and a reduction in NANOG expression, but NANOG expression appears to be slightly higher than POU5F1 expression; moreover, it is more inclined to cytoplasmic expression (Fig. 1B3, 4).

In the next step, we sought to determine the NANOG, POU5F1, and C-MYC expression patterns in embryonic stem cells (Fig. 1C-E). To this end, we used immunocytochemical analysis and observed high expression of NANOG in embryonic stem cells and ES-like cells (Fig. 1C).

### Performance of the Fluidigm real-time RT-PCR

**Fig. 2:**
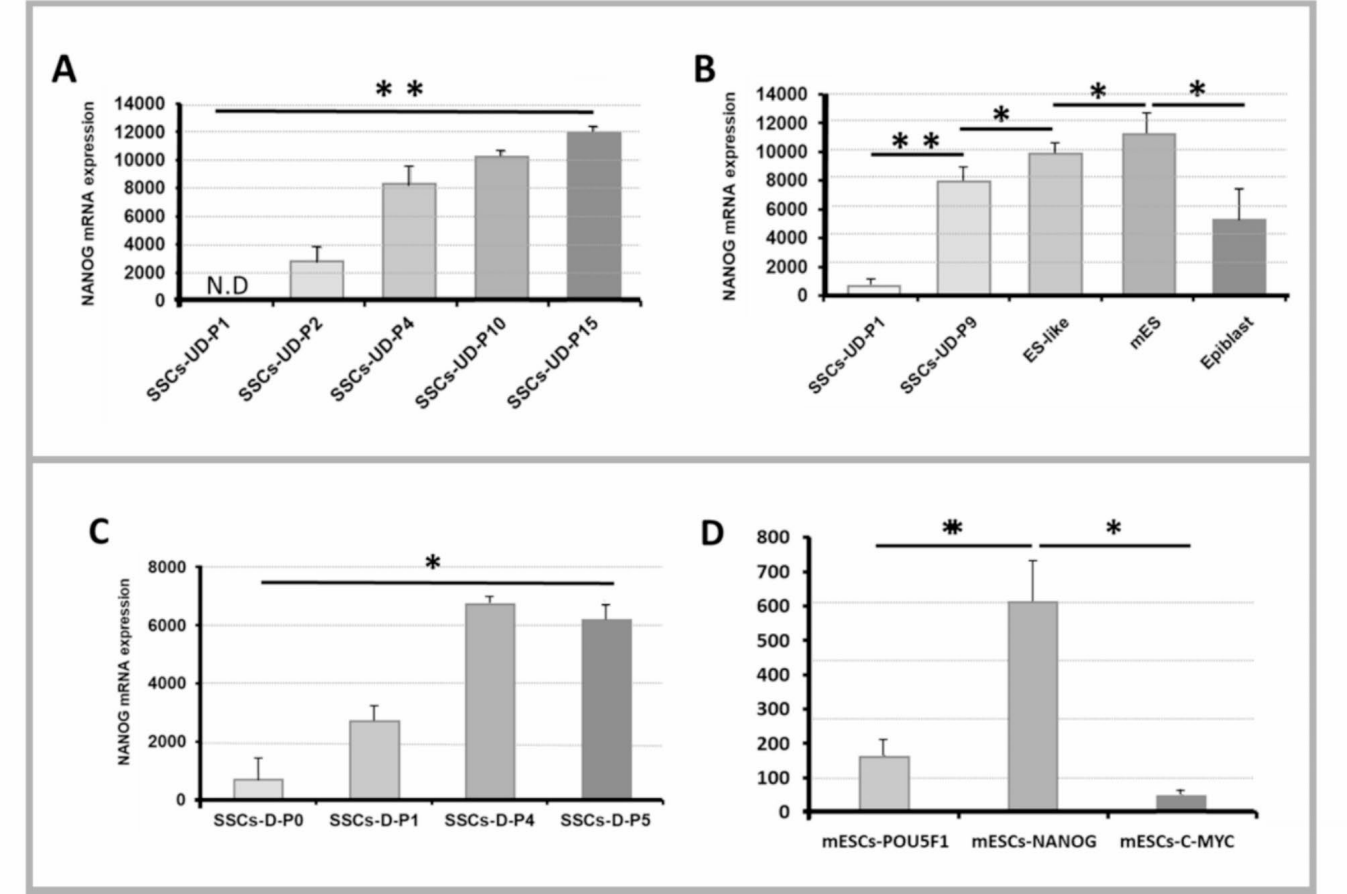
Quantitative mRNA expression by Fluidigm real-time RT-PCR for the NANOG gene (**A**, **B**) and POU5F1, NANOG, and C-MYC in ESCs (**C**). **A** Quantitative mRNA expression by Fluidigm real-time RT-PCR for the NANOG gene in different passages of undifferentiated SSCs. Quantitative mRNA analysis of the NANOG gene has shown that by increasing the passage in undifferentiated SSCs, the mRNA level of NANOG also increases and finally has a significant expression in passage 15 compared to passages 1 and 2. **B** Quantitative mRNA expression by Fluidigm real-time RT-PCR showed that NANOG has High mRNA expression levels in mouse embryonic stem cells and ES-like cells. In contrast, it has lower expression in Epiblast cells, and in P1 undifferentiated SSCs, NANOG has a significantly higher mRNA expression level than P9 undifferentiated SSCs. **C** Quantitative mRNA expression by Fluidigm real-time RT-PCR for the NANOG gene in different passages of differentiated SSCs. mRNA expression analysis of the NANOG gene showed significant expression in P4 and P5 of differentiated SSCs in contrast with P0 and P1, although they do not differ significantly from each other. **D** Quantitative mRNA expression by Fluidigm real-time RT-PCR for NANOG, POU5F1, and C-MYC showed significantly higher expression of NANOG than POU5F1 and C-MYC, respectively, in mouse embryonic stem cells. * *p* ≤ 0.05, ** *p* ≤ 0.01, *** p ≤ 0.001.

Quantitative mRNA expression by Fluidigm real-time RT-PCR for the NANOG gene indicated the mRNA expression level in Passage one and nine of undifferentiated SSCs, ES-like cells, mES, and Epiblast cells. Comparison of NANOG mRNA levels in passages one and 9 of undifferentiated SSCs shows an increase in NANOG expression with the increasing number of passages (Fig. 2A). As shown in figure-1 C, NANOG has a high expression level in mouse embryonic stem cells and ES-like cells, which has also been confirmed in ICC analysis. On the other hand, the decrease in NANOG mRNA expression in epiblast cells compared to embryonic stem cells was also confirmed (Fig. 2B).

Furthermore, we have continued Fluidigm analysis for quantitative investigation of NANOG mRNA expression in different passages of undifferentiated SSCs, which indicated significant expression (*P* < 0.05) of NANOG mRNA in passage 15 and analysis showed that NANOG was not expressed in passage 1. Also, the mRNA level of NANOG increased during each passage compared to previous passages (Fig. 2B).

We also do the same analysis for quantitative investigation of NANOG mRNA expression in different passages of differentiated SSCs (Fig. 2C). The results showed a significant expression (*P* < 0.05) in passage 4 and passage 5 differentiated SSCs in comparison to passages 0 and 1, but passages 4 and 5 did not show a significant difference (Fig. 2C). Also, Quantitative mRNA expression by Fluidigm real-time RT-PCR for NANOG, POU5F1, and C-MYC showed significantly higher expression of NANOG than POU5F1 and C-MYC, respectively, in mESCs (Fig. 2D).

### Stem cell PPI network and sub-network enrichment analyses

**Fig. 3:**
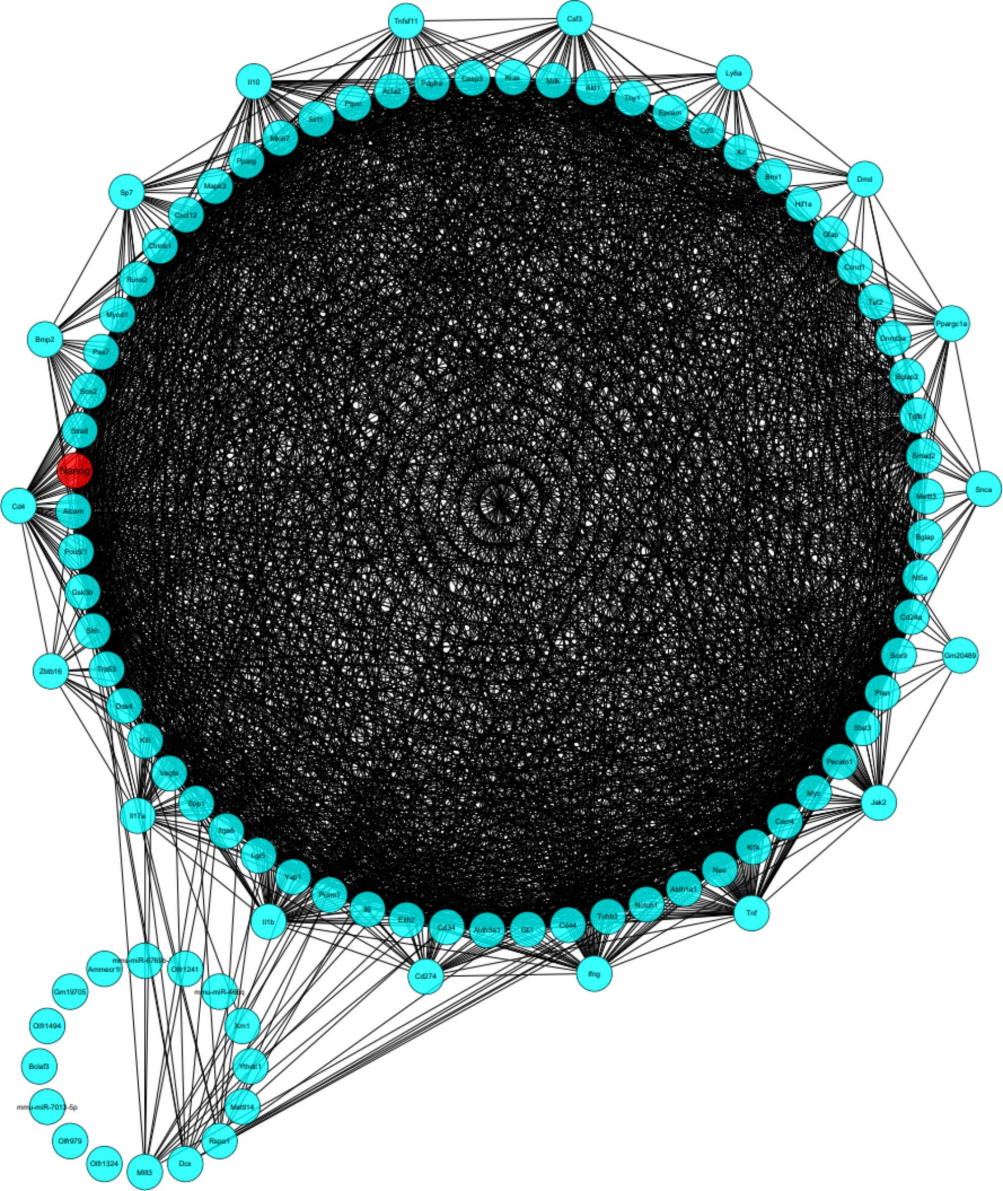
The protein-protein interaction (PPI) Network analysis of stem cell proteins. Cytoscape implemented the network based on the STRING PubMed query. The maximum number of proteins was set as 100 with a confidence score cut-off > 0.40. The red node represents NANOG.

**Fig. 4:**
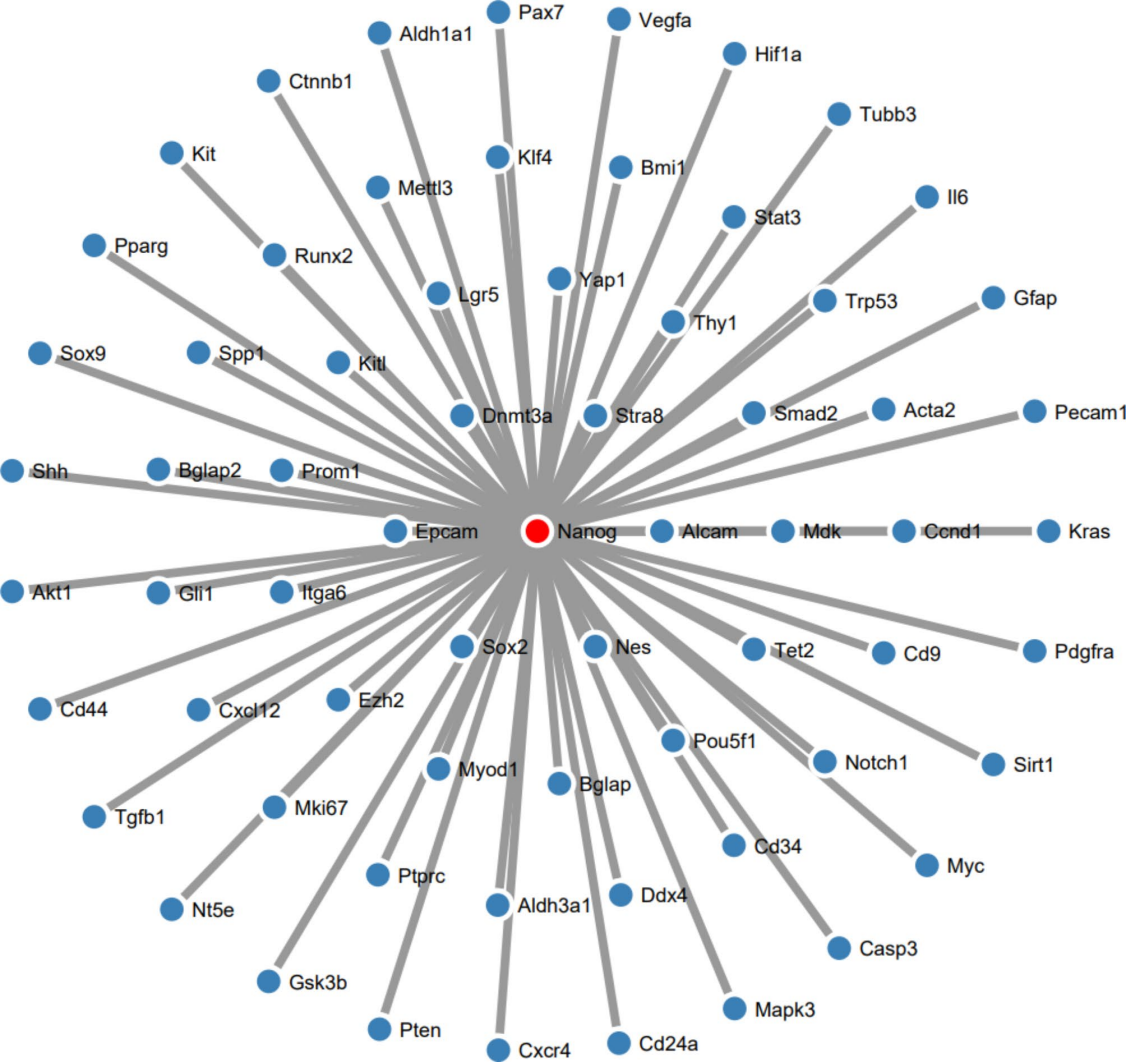
Sub-network consists of interactions of NANOG’s first neighbors’ nodes.

**Fig. 5:**
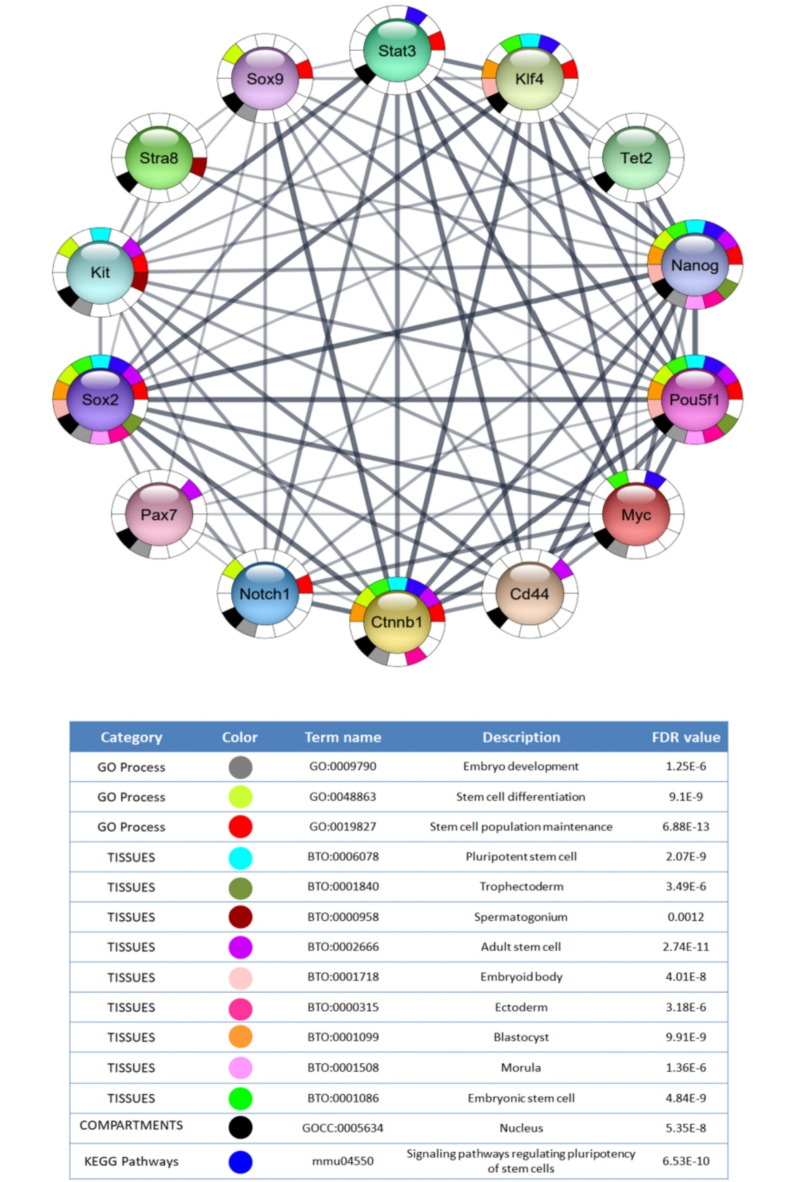
The functional enrichment analysis of selected genes directly interacting with NANOG. Different colored parts of the circles refer to the related biological processes, with line thickness being indicative of evidence strength for a predicted interaction.

**Fig. 6:**
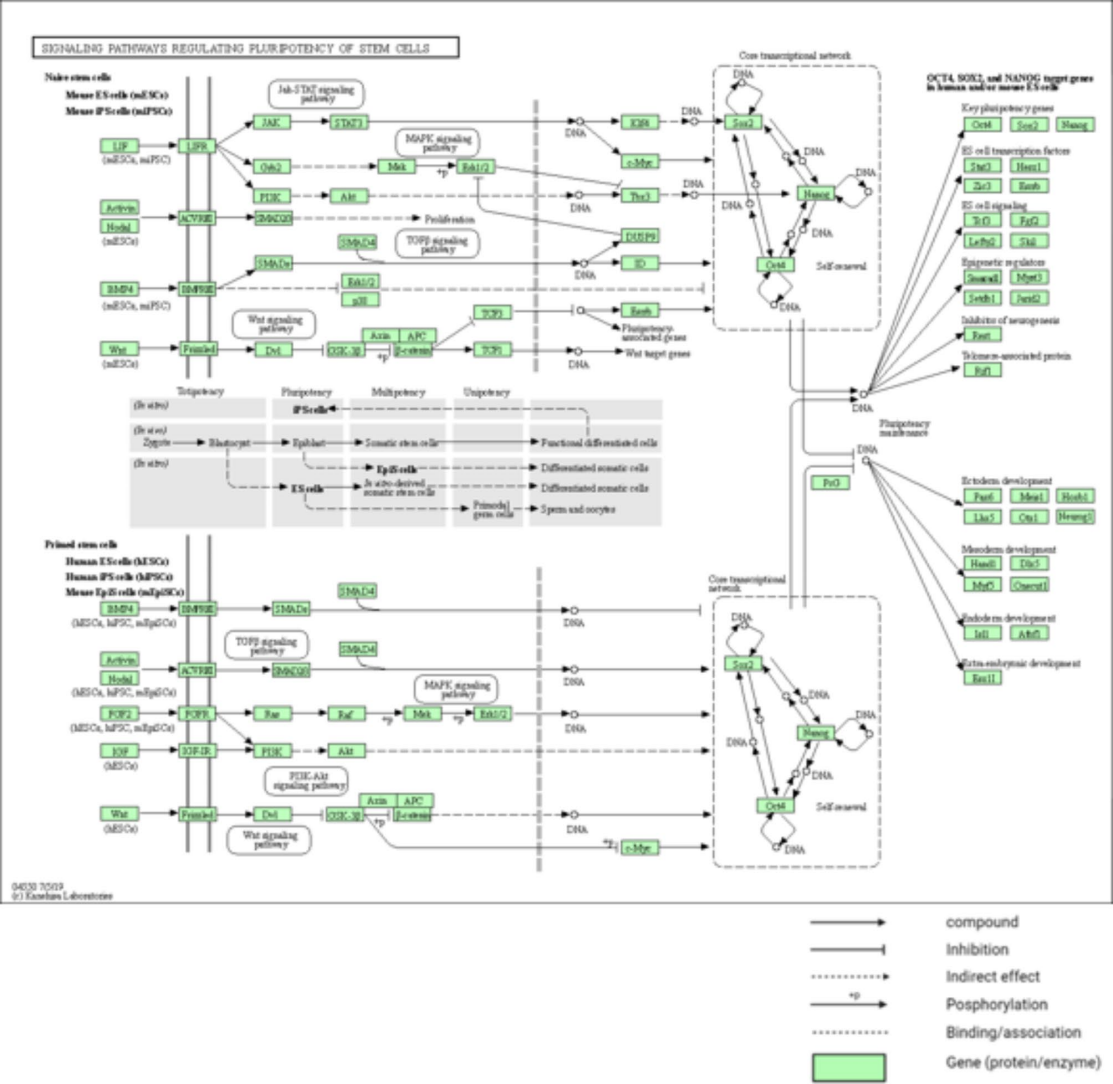
KEGG view on Signaling pathways regulating pluripotency of stem cells in Mus musculus^39^.

There were 100 nodes and 1996 edges in the major component of the “Stem cell” PPI network, which was created using STRING and Cytoscape (Fig. 3). All nodes represent proteins, and edges represent protein-protein associations. According to the network, NANOG is one of the nodes, and it is set in red (Fig. 4). We examined the adjacent nodes with NANOG and produced a sub-network (Fig. 4). This sub-network includes 66 nodes. The implication is that NANOG strongly correlates with other genes associated with stem cells. We conducted STRING Enrichment analysis on 14 randomly chosen genes to assess the molecular functions and cellular locations linked to NANOG (Fig. 5). We have chosen certain biological processes for our experimental studies, focusing on embryo development, differentiation of stem cells, and maintenance of stem cell population. Furthermore, using TISSUES analysis, the positioning of genes in pluripotent stem cells, trophectoderm, spermatogonium, adult stem cell, embryoid body, ectoderm, blastocyst, morula, and embryonic stem cell can be determined. Additionally, through KEGG Pathways, we observed that our genes are involved in the signaling pathways that regulate the pluripotency of stem cells. In addition, using COMPARTMENTS analysis, we also demonstrate our genes’ cellular localization (Fig. 6).

## Discussion

Our study provides unique insights into the expression and regulation of NANOG in various stem cell types, particularly focusing on ES-like cells, Epiblast cells, and embryonic stem cells (ESCs). Recent studies have consistently emphasized the critical role of NANOG in maintaining the pluripotency of ESCs^18^. For instance, Wang et al. (2022) conducted an extensive analysis of NANOG in murine ESCs, highlighting its synergistic interaction with POU5F1 (OCT4) and SOX2 to sustain the undifferentiated state^19^. Their results align with our observations of high NANOG expression in ESCs, reinforcing its established role in pluripotency. However, our study goes beyond simply confirming the role of NANOG in ESCs by examining its expression in ES-like cells and Epiblast cells^20^. Specifically, we observed that NANOG exhibits cytoplasmic localization in ES-like cells, a finding not commonly reported in the literature. This contrasts with most studies that focus on NANOG’s nuclear function. A recent study reported predominantly nuclear localization of NANOG in ESCs, suggesting that its function is largely restricted to transcriptional regulation^21^. The cytoplasmic presence of NANOG in our research suggests potential non-transcriptional roles, possibly in signaling pathways or in regulating the cellular microenvironment, which warrants further investigation. The downregulation of NANOG during differentiation has been a consistent finding across various studies^20–22^. Liu et al. showed that as ESCs transition to Epiblast-like cells, NANOG expression diminishes significantly, marking the loss of pluripotency and the onset of lineage specification^23^ that is in agreement with our findings, where we observed a decrease in NANOG expression in Epiblast cells compared to ESCs^23,24^.

However, our study provides additional nuance by showing that NANOG expression, although decreased, remains higher than POU5F1 in Epiblast cells and is more inclined to cytoplasmic localization^25^. This contrasts with the work of Mora-Castilla et al., who reported a simultaneous reduction in both NANOG and POU5F1 as cells exit the pluripotent state, with no significant distinction between their expression levels^26^. Our findings suggest that NANOG may retain some functionality in the early stages of differentiation, potentially linked to its cytoplasmic presence, which might be involved in preparing cells for differentiation rather than simply marking pluripotency loss^25,26^. Our Fluidigm real-time RT-PCR analysis revealed a progressive increase in NANOG mRNA expression with successive passages in undifferentiated SSCs, contrasting with some reports in the literature. For example, Yu et al. found that prolonged culture of human pluripotent stem cells (hPSCs) often leads to a gradual decrease in NANOG expression, suggesting a decline in pluripotency due to cellular stress or genetic drift over time^27^.

Our results, which show an increase in NANOG expression, could suggest that prolonged culture might enhance pluripotency-related gene expression in certain contexts, particularly in SSCs. This could be due to an adaptation mechanism where SSCs upregulate NANOG to counterbalance differentiation cues or culture-induced stress, a hypothesis that diverges from the trends observed in hPSCs and could reflect species-specific differences or the unique biology of SSCs^27,28^. Furthermore, our findings that differentiated SSCs maintain significant NANOG expression in later passages add a layer of complexity to the understanding of NANOG’s role during differentiation. Studies by Hyslop et al. have reported that NANOG is typically downregulated in differentiated cells. Yet, our results suggest that NANOG may remain actively expressed in certain differentiation contexts, potentially influencing differentiation outcomes or maintaining a degree of plasticity^29^. This highlights a possible divergence in the role of NANOG in SSCs versus ESCs, or it could indicate that the differentiation state is not fully stable and retains some pluripotent characteristics^30^. Integrating NANOG into a broader PPI network and sub-network enrichment analysis provides insights into its interactions with other proteins involved in stem cell regulation^31^. While recent studies like those by Choi et al. have also mapped NANOG’s interaction networks using similar bioinformatics tools, our study is distinguished by its focus on the sub-network associated with NANOG^32^.

Our analysis identified 66 closely interacting proteins, suggesting that NANOG is highly integrated into the broader stem cell regulatory network. This finding contrasts with the work of other studies that identified fewer direct interactions, possibly due to differences in methodological approaches or the specific focus of those studies^33^. For example, the study by Martinez-Sarmiento highlighted key NANOG interactions with factors involved in chromatin remodeling but did not explore broader functional categories^34^. Our enrichment analysis extends this by identifying interactions related to embryo development, stem cell differentiation, and population maintenance. This suggests that NANOG’s role extends beyond simple pluripotency maintenance and orchestrates various developmental processes.

## Conclusion

In summary, our study adds significant depth to the current understanding of NANOG’s role in stem cell biology, particularly in its expression and function across different cell types and during various stages of differentiation. The cytoplasmic localization of NANOG in ES-like and Epiblast cells, the progressive increase in NANOG expression in SSCs, and the detailed PPI network analysis collectively highlight the novel aspects of our findings. These results contrast with or extend the conclusions of recent studies, offering new perspectives on the dynamic roles of NANOG in pluripotency and differentiation. Our research thus provides critical insights that validate existing knowledge and open new avenues for understanding the multifaceted roles of NANOG in stem cells, with implications for both basic research and potential therapeutic applications.

Limitations: Several limitations were encountered in this study that merit discussion. Firstly, the investigation did not utilize FRET (Fluorescence Resonance Energy Transfer) analysis experiments to directly measure the proximity and interaction of NANOG and POU5F1 proteins. This omission primarily stemmed from resource constraints and the need for specialized equipment and expertise. Furthermore, we did not employ co-immunoprecipitation techniques to illustrate the interaction between NANOG and POU5F1 due to the limited availability of suitable antibodies and the complex nature of the interaction. These limitations constrained our ability to comprehensively assess the protein-protein interactions, potentially leaving some aspects of the mechanism unexplored.

## Materials and methods

### Digestion and culture of testicular cells

ES-like cells were generated from 7-week-old C57BL/6 mouse testis. The neonatal mouse was killed, and both testes were removed via a small insecure in the lower part of the abdomen. Mice were sacrificed by carbon dioxide after being used in the experiment. After decapsulation of the testis, tissue was mechanically dissected and dissociated via a two-step mechanical and enzymatic digestion solution that contained 0.05 mg/ml DNase (Sigma Aldrich, USA), 0.5 mg/ml collagenase (Sigma Aldrich, USA), and 0.5 mg/ml dispase (Sigma Aldrich, USA) in an HBSS buffer (PAA, USA) (with shaking and pipetting) at 37˚C for 8 min. Digestion enzymes were stopped with 10% ES cell-qualified FBS and pipetted up and down to obtain a single-cell suspension. After centrifugation, specimens were washed with DMEM/F12, filtered through a 70 μm cell strainer, and centrifuged for 10 min at 1500 rpm. The supernatant was removed, and the suspension of testicular cells was plated onto 0.2% gelatine-coated culture dishes. Cells cultured in the mouse GSC (mGSC) medium consisted of StemPro-34 medium, 1% L-glutamine (PAA, USA), 1% N2-supplement (Invitrogen, USA), 6 mg/ml D + glucose (Sigma Aldrich, USA), 1% penicillin/streptomycin (PAA, USA), 5 µg/ml bovine serum albumin (Sigma Aldrich, USA), 0.1% s-mercaptoethanol (Invitrogen, USA), 30 ng/ml oestradiol (Sigma Aldrich, USA), 60 ng/ml progesterone (Sigma Aldrich, USA), 1% non-essential amino acids (PAA, USA), 10 ng/ml FGF (Sigma Aldrich, USA), 100 U/ml human LIF (Millipore), 1% MEM vitamins (PAA, USA), 8 ng/ml GDNF (Sigma Aldrich, USA), 20 ng/ml epidermal growth factor (EGF, Sigma Aldrich, USA), 30 µg/ml pyruvic acid (Sigma Aldrich, USA), 1% ES cell qualified FBS, 100 µg/ml ascorbic acid (Sigma Aldrich, USA) and 1 µl/ml DL-lactic acid (Sigma Aldrich, USA) with 37 °C and 5% CO2 in air^35,36^.

### Generation of ES-like cells

Neonate mouse testicular cells were cultivated in GS medium. Undifferentiated spermatogonia colonies were manually picked in the primary culture or after the subculture of primary supernatants into the MEF feeder layer. ES-like colonies were generated from undifferentiated spermatogonia cells in a time window between 40 and 125 days after culture initiation. Single cells of ES-like colonies were obtained after trypsinization under mouse ES medium condition consisting of KO-DMEM (or DMEM high-glucose medium), 15% Fetal Bovine Serum (FBS), 1% MEM NEAA solution, 1% L-glutamine, 1% Pen-Strep, 0.1% ß-mercaptoethanol (Sigma Aldrich) and 1000 U/ml Leukemia Inhibitory Factor (LIF; Sigma Aldrich) under MEF feeder layer. ES-like colonies were grown in mESCs media and were passaged every 3–4 days. At the beginning of culture, some undifferentiated spermatogonia cells expressed a low level of Oct4-GFP, but this signal was down-regulated after long-term culture. During the cultivation of undifferentiated spermatogonia cells, we found colonies (rate of 25about 33%) that were similar to mESCs (ES-like cells) or cultured epiblast cells that expressed a high level of Oct4-GFP about 41–125 days after initiation of culture. Generated ES-like cells and mESCs were subcultured in mESCs medium. These cells reached confluence about 4–5 days after initiation of culture. Cells were passaged to a new MEF feeder after washing with PBS and treatment with Trypsin-EDTA for 3 min. Trypsin-EDTA was inactivated with 15% of FBS^37^.

### Immunocytochemical (ICC) staining

Cells were cultured in 24 well plates and fixed with 4% paraformaldehyde. After rinsing with PBS, samples were permeabilized with 0.1% Triton/PBS and blocked with 1% BSA/PBS. After removing the blocking solution, the cells were incubated overnight with primary antibodies. After 29 rinsing, the process was followed by incubation with species-specific secondary antibodies conjugated with different fluorochrome. Labeled cells were counterstained with 0.2 µg/ml DAPI (4’, 6-diamidino 2-phenylindole) for 3 min at room temperature and fixed with Mowiol 4–88 reagent. Each primary antibody in the sample was omitted as a negative control for all markers. Labeled cells were examined with a confocal Zeiss LSM 700 microscope, and images were acquired with a Zeiss LSM-TPMT camera^38^.

### Gene expression analyses on the Fluidigm Biomark system

The expression level of the NANOG gene in SSCs, ESCs, ES-like cells, and Epiblast cells was examined by the Fluidigm Biomark system. Glyceraldehyde-3-phosphate dehydrogenase was utilized as a housekeeping gene for standardization. SSCs, ESCs, ES-like, and Epiblast cells were picked up with a micromanipulator technique, lysed with a solution of lysis buffer that contained 9 µl RT-PreAmp Master Mix (5.0 µl Cells Direct 2× Reaction Mix, Invitrogen, USA), 2.5 µl 0.2× assay pool and 1.3 µl TE buffer, 0.2 µl RT/Taq Superscript III (Invitrogen, USA). Then, the amount of the amplified product of RNA-targeted copies was examined with TaqMan real-time PCR on a BioMark Real-Time Quantitative PCR (qPCR) system. Samples were analyzed in two technical repeats. The Ct values were calculated using Excel and GenEx software^5,38^.

### PPI network construction and analysis

To construct the PPI network, we used the Search Tool for the STRING version 11.5 (Retrieval of Interacting Genes/Proteins database, https://string-db.org/). STRING is a web database that intends to integrate all known and predicted interactions between proteins, including physical interactions and functional associations. STRING app in Cytoscape Software (v 3.8.2) was used to construct the PPI network. The STRING: PubMed query was used as the data source to generate a network among the first 100 proteins in the “Stem cell” in Mus musculus, and the minimum confidence score cut-off was set at 0.40. Subsequently, we identified the first neighbors’ nodes with NANOG and created a sub-network for them.

### Gene enrichment analysis

To investigate the functions of some genes involved in the sub-network and their localization in some tissues, we have performed the STRING Enrichment analysis in the Cytoscape Software (https://cytoscape.org/). We selected 14 genes that are connected to NANOG, including POU5F1, SOX2, SOX9, Pax7, Notch1, Kit, Stat3, Tet2, Stra8, Klf4, Ctnnb1, Cd44, and Myc. We studied several functional enrichments related to our laboratory data without considering a specific FDR value.

### Statistical analysis

The trials were replicated at least 3 times. All groups’ average gene expression was calculated, and the groups were evaluated using one-way analysis of variance (ANOVA), covered by Tukey’s post-hoc tests. The expression of genes was compared with non-parametric Mann-Whitney’s test. The variation between groups was considered statistically reliable if a value of *P* < 0.05 had been acquired. PPI networks were analyzed based on relevant databases or online data analysis tools.

## Data Availability

The data sets analyzed for the current study are available from the corresponding author on reasonable request.

## Abbreviations

SSC: Spermatogonial Stem Cell
ESC: Embryonic Stem Cells
ICM: Inner Cell Mass
mESC: mutant EmbryonicStem Cell
PGC: Primordial Germ Cell
